# Predictors for Development of Asphyxiated Neonates Treated With Therapeutic Hypothermia

**DOI:** 10.1111/apa.17598

**Published:** 2025-01-29

**Authors:** Fabienne Kühne, Nina Wald de Chamorro, Laura Glasmeyer, Maria Grigoryev, Yee Lee Shing, Claudia Buss, Christoph Bührer, Angela M. Kaindl

**Affiliations:** ^1^ Center for Chronically Sick Children Charité – Universitätsmedizin Berlin Berlin Germany; ^2^ Department of Neonatology Charité – Universitätsmedizin Berlin Berlin Germany; ^3^ Department of Pediatric Neurology Charité – Universitätsmedizin Berlin Berlin Germany; ^4^ German Center for Child and Adolescent Health (DZKJ), partner site Berlin Berlin Germany; ^5^ German Heart Center Charité – Universitätsmedizin Berlin Berlin Germany; ^6^ Institute of Neuroradiology, Charité – Universitätsmedizin Berlin Berlin Germany; ^7^ Department of Psychology Goethe Universität Frankfurt am Main Frankfurt Germany; ^8^ Institute for Medical Psychology, Charité – Universitätsmedizin Berlin Berlin Germany; ^9^ Department of Pediatrics, Development, Health and Disease Research Program University of California Irvine California USA; ^10^ Institute for Cell Biology and Neurobiology, Charité – Universitätsmedizin Berlin Berlin Germany

**Keywords:** asphyxia, development, epilepsy, hypothermia

## Abstract

**Aim:**

To describe the long‐term neurodevelopmental outcomes of asphyxiated neonates treated with hypothermia in association with neonatal magnetic resonance imaging (MRI) findings.

**Methods:**

We evaluated, retrospectively, clinical and radiological single‐centre data at 0, 2, and 5 years of age of 53 asphyxiated neonates born between 2005 and 2015. Neonatal cranial MRI was re‐evaluated using the Weeke score ranging from 0 (normal finding) to 55 (cerebral devastation) by a single neuroradiologist blinded to patient outcomes. Neurodevelopmental outcomes were evaluated using the Bayley Scales of Infant Development (BSID) at 2 years, and tests assessing intellectual performance at 5 years of age.

**Results:**

Of the 191 asphyxiated neonates treated with hypothermia, 53 returned for their 5‐year follow‐up. There were 10 children with MRI scores ≥ 10, all of whom had epilepsy, 9 had severe cognitive impairment, and 9 had cerebral palsy. In contrast, MRI scores < 10 were poorly predictive of later development. BSID at 2 years of age showed good correlation with IQ scores at 5 years of age (*R*_*s*_ = 0.58, *p* < 0.001).

**Conclusion:**

The Weeke score can be used to identify severely impaired children in the neonatal period. In contrast, the neurocognitive test results at 2 years of age were indicative of mild or moderate impairment at 5 years of age.

**Abbreviations**
aEEG: *amplitude‐integrated electroencephalography*
AUC: area under the curve
BSID: Bayley Scales of Infant Development
BUEVA: Basisdiagnostik Umschriebener Entwicklungsstörungen im Vorschulalter
CI: confidence interval
CP: cerebral palsy
DWI: diffusion‐weighted imaging
GMFCS: gross motor function classification system
HIE: hypoxic–ischaemic encephalopathy
IQ: intelligence quotient
K‐ABC: Kaufman‐ABC
MDI: Mental Development Index
MRI: magnetic resonance imaging
ROC: receiver operating characteristic
*R*_*s*_
: Spearman rank‐order coefficients
SD: standard deviation
SON‐R: Snijders‐Oomen non‐verbal intelligence test
WPPSI: Wechsler Preschool and Primary Scale of Intelligence

**Summary**

- Trials assessing induced hypothermia aim at neurodevelopmental at 2 years of age, but problems arising from asphyxia may emerge later.
- Severe but not subtle impairment at 5 years is picked up by early magnetic resonance imaging, whereas the results of the standardised test at 2 and 5 years are strongly related.
- Children with encephalopathy should be closely followed also when imaging does not indicate brain damage.

## 1 Introduction

Therapeutic hypothermia was introduced at the beginning of the millennium to mitigate neurological sequelae in infants with perinatal asphyxia. In several large randomised controlled trials, therapeutic hypothermia was demonstrated to reduce rates of death and severe disability in children affected by moderate or severe hypoxic–ischaemic encephalopathy (HIE) [1, 2, 3, 4]. Therapeutic hypothermia has become the standard of care for asphyxiated newborns in highly developed countries [5, 6, 7, 8].

Despite therapeutic hypothermia, HIE after perinatal asphyxia is one of the most frequent causes for paediatric morbidity and mortality [9]. The mortality rate of HIE remains at 10%–20% [10, 11], and around 20%–30% of survivors have cognitive impairments, poor executive functioning, and attention disorders. Approximately 50%–60% of children exhibit normal intelligence levels, while around 5%–10% display severe disabilities requiring continued support [1, 10, 11, 12, 13]. The incidence of cerebral palsy (CP) among children treated with therapeutic hypothermia remains significant. Pekeles et al. [14] found that 8% of cooled neonates developed CP, with a higher proportion of severe cases.

When assessed at early school age, children who underwent hypothermia for perinatal asphyxia may have decreased intelligence quotient (IQ) scores, lower language scores, and higher rates of minor neurological dysfunction even in the absence of CP [15, 16, 17]. Moreover, executive function difficulties and other subtle cognitive deficits may not be apparent at early school age but emerge during adolescence [10, 18]. More than 25% of children with childhood outcomes considered favourable may have emerging deficits in early adolescence, including increased executive difficulties and motor coordination issues [10, 19].

It is important to identify predictors for later development at an early stage to guide targeted support and counselling. Known predictors for outcomes after HIE and treatment with therapeutic hypothermia include several clinical, electroencephalographic, and imaging variables. Poorer outcomes are associated with a low 5‐min Apgar score, severe acidosis or high lactate levels, severely abnormal neurological status, and abnormal amplitude‐integrated electroencephalography (aEEG) within the first 48 h. Furthermore, the necessity of respiratory support or the administration of epinephrine were found to be significant indicators of adverse outcomes [5, 13, 20, 21, 22].

Several studies have reported that neonatal cranial magnetic resonance imaging (MRI) data to function as a predictor for long‐term neurologic outcome, especially abnormalities in the deep grey matter regions [23, 24, 25, 26, 27, 28, 29, 30, 31].

Abnormal EEG and MRI findings have high specificity and positive predictive value for predicting adverse outcomes. However, their negative predictive value is lower, indicating that normal findings do not guarantee a favourable outcome [13, 26].

To address this limitation of qualitative evaluation of MRI images, Weeke et al. [26] developed a novel MRI score that can be obtained from routine clinical scans in the first week of life, aimed at predicting poor outcome. This score includes diffusion‐weighted imaging (DWI) scans and includes more subtle abnormalities than the established Barkovich and NICHD scores [32].

Here, we aimed to evaluate the predictive value of the neonatal Weeke MRI score for neurodevelopmental status at 5 years of age. Whereas Weeke et al. divide outcome into normal and abnormal development in a dichotomous fashion, we were interested in whether the score may indicate the degree of impairment, as it also takes subtle changes in MRI images into account. Furthermore, we aimed to examine to what extent cognitive development at 5 years of age is heralded by low scores of the Bayley Scales of Infants Development at 2 years of age [22, 33] in children with perinatal HIE. The overarching aim of the current study was to evaluate neonatal radiological and infant neurocognitive predictors for preschool neurodevelopmental status in children following therapeutic hypothermia treatment for HIE.

## 2 Methods

### 2.1 Participants

We performed a retrospective cross‐sectional study based on clinical data and cranial MRI scans of 53 children that were admitted to Charité—Universitätsmedizin Berlin, Germany, between 2005 and 2015 for HIE who received therapeutic hypothermia. Overall, 191 infants were treated with therapeutic hypothermia between 2005 and 2015; 23 died in the neonatal period. In the first 2 years, induced hypothermia was used only within the framework of a randomised controlled trial, and only two surviving infants had been assigned to hypothermia. Standardised neurodevelopmental data at 5 years of age were available only in a fraction of children (Figure S1). Of these children, 47 received a neonatal MRI scan, 45 attended the 2‐year follow‐up assessment, and 40 children underwent both examinations (2‐ and 5‐year follow‐up). This study was approved by the local ethics committee (approval No. EA2/106/20).

Neonates born < 36 weeks of gestation and those with chromosomal anomalies were excluded from the study. HIE was defined as a condition with seizures and/or pathological aEEG and/or abnormal level of consciousness in combination with at least one additional neurologic symptom, such as muscular hypo/hypertonia or missing/abnormal reflexes. Perinatal asphyxia was defined as a 10‐min Apgar score ≤ 5, pH ≤ 7.00, base deficit ≥ 16 mmol/L, and/or resuscitation 10 min after birth. Cooling was initiated as soon as possible within the first 6 h of life and continued for 72 h at 33°C–34°C. After 72 h, the infants were rewarmed gradually over 6–8 h.

Given the retrospective nature of the study and birth constellations, not all data points were available for all infants. Especially for home deliveries, values for pH or base deficit in the first 60 min of life were not available, with paper‐based records partially missing.

### 2.2 MRI Score

In the first week of life (days 4–6), cranial MRI was performed as part of routine clinical care. MRI scans included conventional T1‐weighted, T2‐weighted, and DWI sequences without the injection of contrast material at 1.5 T, the exception being few images at 3 T in 2015. Images were collected at various scanners, depending on the year of birth and treatment site.

A single neuroradiologist, who was blinded to the clinical outcome, reviewed all images for quality and classified the lesions according to the score reported by Weeke et al. [26] This score evaluates brain lesions in three brain areas and assigns a score to each region for the extent of injury: (i) deep grey matter (thalamus, basal ganglia, posterior limb of the internal capsule, brainstem, perirolandic cortex, hippocampus) with a maximum subscore of 23; (ii) cerebral white matter/cortex (cortex, cerebral white matter, optic radiations, corpus callosum, punctate white matter lesions, parenchymal haemorrhage) with a maximum subscore of 21; (iii) cerebellum subscore (cerebellum and cerebellar haemorrhage) with a maximum subscore of 8; and (iv) additional score (intraventricular or subdural haemorrhage and sinovenous thrombosis) with a maximum subscore of 3. The total score was obtained by adding the four subscores. A score of 55 was, thus, the maximum score for the maximum extent of brain damage.

### 2.3 Neurodevelopmental Outcomes

After discharge, all patients were referred to the Center for Chronically Sick Children at Charité—Universitätsmedizin Berlin for regular follow‐up visits at 2 and 5 years of age. The Bayley Scales of Infant Development (BSID) Mental Development Index (MDI) was used to evaluate neurocognitive outcomes at 2 years of age. IQ testing was part of the routine assessment during the 5‐year follow‐up. By default, the Kaufman‐ABC version II (K‐ABC) was used for this age group. In few cases, the Wechsler Preschool and Primary Scale of Intelligence (WPPSI) or BUEVA version III (Basisdiagnostik Umschriebener Entwicklungsstörungen im Vorschulalter). For children who did not speak and understand German properly, the Snijders‐Oomen non‐verbal intelligence test (SON‐R) was used. All tests provided standardised scores, with test results between 85 and 115 indicating a normal IQ, scores ≥ 115 representing an above‐average IQ, and scores < 85 a below‐average IQ.

In addition, we recorded the diagnoses of epilepsy and CP at 5 years of age. The severity of CP was categorised according to the Gross Motor Function Classification System (GMFCS). For infants with severe CP (GMFCS IV or V) and severe global developmental delay who could not be formally tested with BSID or K‐ABC, scores between 36 and 45 were randomly assigned. For testable children, we evaluated the presence of motor/coordination disorders, behavioural/psychiatric disorders, or a speech disorder at 5 years of age.

### 2.4 Statistical Analyses

Data were collected in REDCap (https://www.project‐redcap.org). SPSS 25 (IBM Corporation, Armonk, NY) was used to calculate descriptive statistics and to generate receiver operating characteristic (ROC) curves. Descriptive statistics were expressed as frequencies and percentages of the cohort studied. The median and minimum‐maximum values were determined as appropriate. Correlations of IQ score at 5 years of age with total neonatal MRI‐based Weeke scores and BSID MDI were evaluated by Spearman rank‐order coefficients (*R*_*s*_). ROC curves for typical development at 5 years of age, defined as an IQ > 85, BSID > 85, no CP, no epilepsy, and no motor/coordination, behavioural/psychiatric, or speech disorders, were plotted for the BSID score at 2 years of age and total MRI score, respectively. We calculated the area under the curve (AUC) with 95% confidence intervals (CIs) with nonparametric standard errors and used the maximum sum of sensitivity and specificity to determine the cut‐off for further analysis. Two‐sided *p* values *p* < 0.05 were considered significant.

## 3 Results

### 3.1 Patient Characteristics and General Findings

The 53 children assessed for this study were representative for the whole population of surviving infants regarding gestational age, sex, birthweight, and other neonatal characteristics (see Table S1). Treatment started on average at the age of 3 h 48 min (median 3 h), and was commenced within 6 h after birth in 89.8% of infants.

Neurodevelopmental outcomes at the 5‐year follow‐up assessment are summarised in Table S1. Median age at the visit was 5 years and 5 months. A total of 37 children were tested using K‐ABC, three children using WPPSI, two using SON‐R, and one child was tested with BUEVA. The median IQ score of testable children was within the normal range at 103. Twenty‐four children (45.3%) had an IQ in the normal range (85–114), 11 (20.8%) of whom scored in the lower normal range (85–99), and 13 (24.5%) in the upper normal range (100–114). Nine children (17.0%) scored above average (≥ 115), and 10 children (18.9%) scored below average (60–84). The remaining 10 children (18.9%) were globally impaired and could not participate in any standardised IQ tests. They were assigned scores between 36 and 45 randomly for graphical representation; their scores were not included in the statistical analyses. The Kolmogorov–Smirnov test for the IQ scores at 5 years indicated that data were not normally distributed (*p* = 0.024). All globally impaired children developed epilepsy, and all but one child developed CP GMFCS II‐V.

At 5 years of age, 15 of the 43 children tested (34.9%) were diagnosed with motor/coordination, behavioural/psychiatric, or speech disorders (Table 1). The incidence for these three disorders was approximately equally distributed, with two or more disorders occurring in about half of the children.

**TABLE 1:** Neurodevelopmental outcomes at 2 and 5 years‐of‐age in children with concomitant disorders (*n* = 15).

Epilepsy at 5 years	CP at 5 years	Motor/coordination disorder at 5 years	Psychiatric/behavioural disorder at 5 years	Speech disorder at 5 years	MRI score at 4–5 days	BSID MDI at 2 years	IQ at 5 years
		x	x		—	114	118
				x	0	81	111
				x	4	78	108
		x	x	x	—	—	104
		x	x		1	—	99
			x		7	110	99
		x			6	88	98
			x	x	9	88	96
				x	0	—	89
		x			9	—	89
x	x			x	9	68	82
		x			0	—	82
x	x			x	25	—	80
			x		4	68	80
		x		x	0	92	76

### 3.2 MRI Score and Neurocognitive Development

MRI data were available for 47 of the 53 children for re‐evaluation and scoring. MRI data are displayed in Table 2. MRI scans were performed within the target range of days 4–6 of life in 36/47 of the cases. Overall, median MRI scores were within the lower range (meaning low‐grade brain damage). Almost half of the children (46.8%, *n* = 22) had no white and grey matter lesions, and 13 of these children (27.7%) even showed no anomalies, resulting in a total MRI score of 0. MRI scans of 14 infants (29.8%) revealed either grey or white matter lesions; all of these children had a total MRI score < 10. MRI scans of 11 children (23.4%) displayed grey and white matter lesions, 10 of them were diagnosed with extensive brain damage with a total MRI score ≥ 10. One infant with a score ≥ 10 showed additional MRI anomalies (genetic pontocerebellar hypoplasia), which could be at least partly responsible for the severity of impairment.

**TABLE 2:** MRI characteristics of cohort.

Parameter	Median	Mean	Min	Max	*n*
Age at MRI (days)	5.5	7.18	4	42	48
Total score	3.0	6.78	0	34	47
Grey matter subscore	0.0	3.65	0	21	47
White matter subscore	0.0	2.36	0	17	47
Cerebellum subscore	0.0	0.17	0	2	47
Additional subscore	1.0	0.59	0	2	47

**FIGURE 1:**
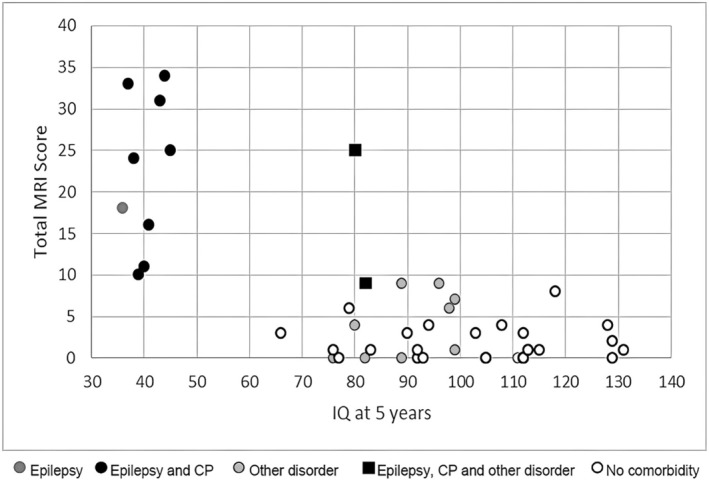
IQ at 5 years and total MRI score. Correlation of total MRI score and IQ at 5 years of age is shown in Figure 1. There was no significant correlation between total MRI score and IQ > 60 (*R*_*s*_ = −0.122, *p* = 0.47, *n* = 38). All but one child (*n* = 9) with a total MRI score ≥ 10 belonged to the group of severely impaired children and were randomly assigned an IQ score between 36 and 45 for graphical representation. Developmental disorders, coordination disorders, psychiatric abnormalities, and visual and hearing disorders were summarised under ‘other’.

The correlation of the total MRI score and the IQ at 5 years of age is depicted in Figure 1. Overall, there was no significant correlation between the total MRI score and an IQ > 60 (*R*_*s*_ = −0.122, *p* = 0.47, *n* = 38). ROC curves for typical development at 5 years of age were plotted for the total MRI score. The AUC was 0.43 with 95% CI 0.21–0.65 (*p* = 0.52). The sum of sensitivity and specificity of the ROC curve peaked at a Weeke score of 10 (> 9.5), which is close to previous results of Weeke et al. [26]. Nine of the ten children with an MRI score ≥ 10 displayed severe global impairment that prevented IQ measurements with standardised tests. Moreover, all children with an MRI score ≥ 10 developed epilepsy, and all but one had CP. One child who developed epilepsy and CP with an MRI score of 9 had suffered from an extensive neonatal left middle cerebral artery infarction in addition to hypoxia, which was likely a contributing factor to these comorbidities.

Children with an MRI score between 5 and 10 were likely to have an IQ < 100. Only one child with an MRI score of 8 had an IQ > 100. However, in this case, the high MRI score was not driven by high scores of grey or white matter damage, but by cerebellar and intraventricular haemorrhage.

For children with an MRI score between 0 and 4, no prediction about further neurocognitive development could be made, except that IQ was in the testable range as there were children with a normal MRI whose IQ ranged between 76 and 129. The MRI score did not allow for the prediction of motor/coordination, behavioural/psychiatric, or speech disorders at 5 years of age.

### 3.3 Correlation Between 2‐ and 5‐Year Outcomes

Next, we analysed if cognitive outcome at 2 years of age could be used as a predictor for IQ at 5 years of age. Cognitive 2‐year outcomes obtained by BSID MDI are summarised in Table 3. Of the 43 children tested at 5 years of age, 36 also participated in BSID MDI testing 3 years earlier with a median age of 24 months, and one child was tested with Griffiths Scales of Development. They obtained a median score of 104 within the normal range. The distribution of BSID MDI scores was similar to IQ scores at 5 years of age, with 52.3% having a BSID MDI score within the normal range; 11.4% being below average and 15.9% being above average. Eight children (18.2%) were severely impaired at 2 years of age and could not be tested.

**TABLE 3:** Testing characteristics at 2‐year follow‐up.

Parameter	
Age at follow‐up (months), median (min–max)	25 (21–33)
BSID score, median (min–max)	104 (68–131)
BSID score, mean (SD)	100 (16.2)
BSID ≥ 115, *n* (%)	8 (18.2)
BSID 85–114, *n* (%)	23 (52.3)
BSID 60–84, *n* (%)	5 (11.4)
Severely impaired (not testable), *n* (%)	8 (18.2)

**FIGURE 2:**
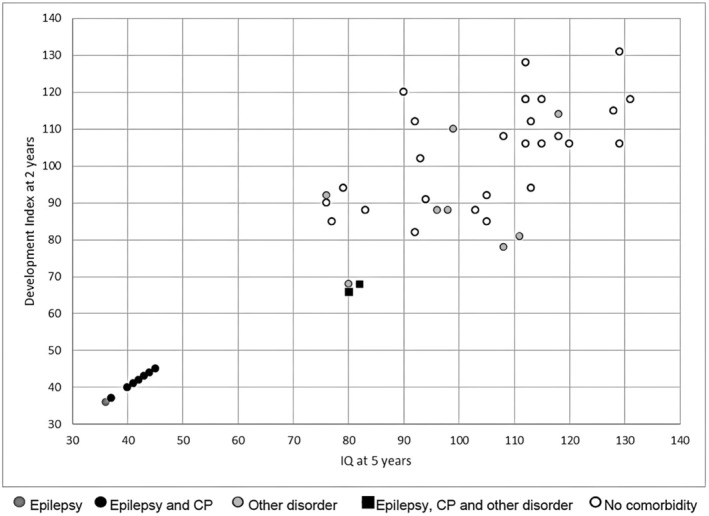
BSID at 2 years and IQ at 5 years. The correlation between BSID at the second‐year follow‐up and IQ at the 5‐year follow‐up is depicted in Figure 2. There was a strong and significant correlation between 2‐ and 5‐year‐cognitive outcomes (*R*_*s*_ = 0.58, *p* = 0.000, *n* = 36) for children with IQ > 50. All children who could not be tested at 2 years of age due to severe impairment could also not be tested at 5 years of age and were randomly assigned BSID and IQ scores between 36 and 45 for graphical representation. Developmental disorders, coordination disorders, psychiatric abnormalities, and visual and hearing disorders were summarised under ‘other’.

The relationship between BSID MDI and IQ scores at 5 years of age is displayed in Figure 2. We identified a significant positive correlation (*R*_*s*_ = 0.58, *p* < 0.001, *n* = 36) between the BSID MDI score at 2 and the IQ score at 5 years of age. This result did not change substantially when excluding children with IQ tests other than K‐ABC (*R*_*s*_ = 0.56, *p* = 0.001, *n* = 33). ROC curves for typical development at 5 years of age were plotted for the BSID MDI score at 2 years of age. The AUC was 0.83 with 95% CI 0.70–0.97 (*p* = 0.007). Hence, in contrast to the total MRI score, neurocognitive testing at 2 years of age could be used to predict cognitive outcomes in later life. All children with a BSID score > 94 reached an IQ in the normal range at 5 years of age. Another finding was that severe impairment at 2 years of age was highly predictive of severe impairment at 5 years of age; all children who were classified as not testable due to global impairment at 2 years of age were also not able to perform an IQ test at 5 years of age.

Table 1 indicates that 73% (11 out of 15) children with a motor/coordination, behavioural/psychiatric, and/or speech disorders reached an IQ score < 100, with 33% of them being < 85. Ten of the 15 children with a disorder participated in the 2‐year follow‐up as well. Eight achieved a BSID MDI < 100, 5 of them scored < 85.

## 4 Discussion

We analysed two predictors for development at preschool age of asphyxiated neonates treated with therapeutic hypothermia: (1) brain MRI results in the first week of life and (2) neurocognitive testing at the age of 2 years.

The Weeke score and the BSID were chosen as predictors due to their validated predictive value and complementary strengths in assessing long‐term prognosis. The Weeke score provides an immediate, comprehensive evaluation of MRI directly after the acute phase of treatment, while the BSID offers tracking developmental progress over time. Together, the integration of both, immediate and long‐term assessment tool offer a robust, multidimensional approach to predict outcomes, allowing healthcare providers customise treatment in the long‐term care of affected infants and improve parents counselling [13, 17, 26, 32].

We analysed to what extent the brain MRI after birth can predict neurocognitive development at 5 years of age. We found that the MRI can be used as a predictor for severely adverse outcomes. In line with the results of Weeke et al. [26] MRI scores of 10 or more were strongly associated with extremely dismal outcomes. However, moderate abnormal MRI findings were poorly predictive for mild to moderate impairment at 5 years of age. This mirrors findings from asphyxiated infants enrolled in the High‐Dose Erythropoietin for Asphyxia and Encephalopathy (HEAL) trial who underwent neonatal MRI at 4–6 days of life. Data of the HEAL trial showed a strong association between severe brain injury on MRI with severe neurodevelopmental impairment at 2 years of age, while infants had similar neurodevelopmental outcomes if they had normal, mild, or moderate MRI scores [30].

There are several explanatory approaches for this result. First, there may be changes caused by injuries due to HIE, which are not picked up by MRI investigations performed in the first days after asphyxia. Brain injury after HIE has been divided into four phases (acute, secondary, latent, and tertiary phase). The secondary and latent phases in the first hours to days after HIE is characterised by oxidative stress and inflammation, which can lead to cell death. They are followed by a tertiary phase, which can last months to years and is characterised by remodelling and late cell death [34, 35]. Typically developed and mildly or moderately impaired children may differ in processes at the cellular level of the latent and secondary phases that are not detectable with standard diagnostic imaging [30]. Second, after the neonatal period, no further routine diagnostic (biochemical markers, imaging) other than neurocognitive testing has been performed to reveal damage from the tertiary period that may explain the differences between these two groups. Hence, future research could address this research gap by longitudinal assessment of children with perinatal asphyxia with more comprehensive neurodevelopmental assessments, including multimodal MRI scans, cognitive assessments across different domains, and biochemical measures. Third, other factors may exist in childhood that led to different neurocognitive outcomes despite the same initial clinical conditions. Especially socio‐economic background and family environment may play an important role, as also shown in the HEAL study [31]. In studies investigating extremely preterm infants or children recovering from traumatic brain injuries, a favourable family environment and a higher socio‐economic status were found to be strongly associated with better neurocognitive outcomes [36, 37]. A study by Bartha‐Doering et al. [38] even found that for recovery after arterial ischaemic stroke, socio‐economic status is a stronger predictor than clinical factors. A limitation of our study is that it did not take into account the socio‐economic status, an issue to be addressed in future research.

We next investigated to what extent the BSID MDI scores assessed at 2 years of age are a predictor for IQ at 5 years of age. We found that neurocognitive impairment at age 5 years can be predicted at the age of 2 years, with a strong correlation between cognitive outcomes at 2 years and at preschool age, This is consistent with other studies from this area of research [22, 33]. Moreover, children with a BSID MDI score < 100 at the 2‐year follow‐up were more likely to develop motor/coordination, behavioural/psychiatric or speech disorders. Hence, children with a below‐average BSID MDI at 2 years of age should be monitored carefully and be directed to adequate therapies if needed. Children with motor/coordination, behavioural/psychiatric, or speech disorders mostly had MDI scores at 2 years, as well as IQ scores at 5 years of age, in the lower normal range or below normal range, but larger studies are needed for a more detailed analysis.

We observed that the IQ distribution at 5 years of age differed from a population of the same age. The average IQ of testable children was about five points below that of typically developed comparison groups from the United Kingdom and Ireland [39]. The actual difference was even larger because our calculation did not include severely impaired children who could not be tested. Moreover, the IQ distribution of our cohort was not normally distributed, as there was a second peak at the lower end, representing the children with severe impairment. Similar observations (mean IQ in the low normal range, greater share of impaired infants) were made for children born very preterm [39, 40, 41] and children with congenital heart disease [42, 43]. When evaluating and interpreting IQ scores, the secular rise of IQ test results (Flynn effect) must also be considered [44]. To date, the expected IQ deviations in children with disabilities compared to their healthy peers are likely to be more pronounced, as the Flynn effect does not equally impact both groups [45].

In summary, we report the MRI score developed by Weeke et al. [26] to be a powerful tool for predicting severe impairment, but the MRI examinations apparently fail to identify mildly or moderately impaired children. Therefore, it is important to follow‐up asphyxiated children regularly after discharge. Since neurodevelopmental problems in later life are already apparent through neurocognitive testing at the age of 2 years and may be diagnosed clinically prior to that age, especially in the severely affected, treatments can be initiated early on. Nevertheless, more research needs to identify predictors that distinguish even earlier between normally developed and mildly or moderately impaired infants to provide the latter group with adequate support at the earliest possible stage.

## Author Contributions

**Fabienne Kühne:** writing – review and editing, writing – original draft, formal analysis, software, project administration, investigation, validation, visualization, data curation. **Nina Wald de Chamorro:** writing – review and editing, writing – original draft, formal analysis, software, project administration, investigation, visualization, validation, conceptualization, data curation, resources, methodology. **Laura Glasmeyer:** writing – review and editing, writing – original draft, investigation, data curation, formal analysis. **Maria Grigoryev:** writing – review and editing, formal analysis, investigation, validation, software. **Yee Lee Shing:** writing – review and editing, data curation, conceptualization, investigation. **Claudia Buss:** writing – review and editing, data curation, conceptualization, investigation. **Christoph Bührer:** project administration, conceptualization, methodology, writing – review and editing, writing – original draft, funding acquisition, supervision, investigation. **Angela M. Kaindl:** project administration, conceptualization, methodology, writing – review and editing, writing – original draft, funding acquisition, supervision, investigation.

## Conflicts of Interest

The authors declare no conflicts of interest.

## Supporting information


Figure S1.

Table S1.
Table S2.

## References

[1] P. D. Gluckman , J. S. Wyatt , D. Azzopardi , et al., “Selective Head Cooling With Mild Systemic Hypothermia After Neonatal Encephalopathy: Multicentre Randomised Trial,” Lancet 365, no. 9460 (2005): 663–670. 15721471 10.1016/S0140-6736(05)17946-X

[2] S. Shankaran , A. R. Laptook , R. A. Ehrenkranz , et al., “Whole‐Body Hypothermia for Neonates With Hypoxic–Ischemic Encephalopathy,” New England Journal of Medicine 353, no. 15 (2005): 1574–1584. 16221780 10.1056/NEJMcps050929

[3] D. Azzopardi , B. Strohm , N. Marlow , et al., “Effects of Hypothermia for Perinatal Asphyxia on Childhood Outcomes,” New England Journal of Medicine 371, no. 2 (2014): 140–149. 25006720 10.1056/NEJMoa1315788

[4] I. Guidotti , L. Lugli , M. P. Guerra , et al., “Hypothermia Reduces Seizure Burden and Improves Neurological Outcome in Severe Hypoxic‐Ischemic Encephalopathy: An Observational Study,” Developmental Medicine and Child Neurology 58, no. 12 (2016): 1235–1241. 27444888 10.1111/dmcn.13195

[5] S. Shankaran , A. Pappas , S. A. McDonald , et al., “Childhood Outcomes After Hypothermia for Neonatal Encephalopathy,” New England Journal of Medicine 366, no. 22 (2012a): 2085–2092. 22646631 10.1056/NEJMoa1112066 PMC3459579

[6] S. E. Jacobs , M. Berg , R. Hunt , W. O. Tarnow‐Mordi , T. E. Inder , and P. G. Davis , “Cooling for Newborns With Hypoxic Ischaemic Encephalopathy,” Cochrane Database of Systematic Reviews (2013): CD003311, 10.1002/14651858.CD003311.pub3. 14583966

[7] F. Groenendaal , A. Casaer , K. P. Dijkman , et al., “Introduction of Hypothermia for Neonates With Perinatal Asphyxia in The Netherlands and Flanders,” Neonatology 104, no. 1 (2013): 15–21. 23615314 10.1159/000348823

[8] G. Simbruner , R. A. Mittal , F. Rohlmann , R. Muche , and neo.nEURO.network Trial Participants , “Systemic Hypothermia After Neonatal Encephalopathy: Outcomes of neo.nEURO.network RCT,” Pediatrics 126, no. 4 (2010): e771–e778. 20855387 10.1542/peds.2009-2441

[9] J. E. Lawn , S. Cousens , and J. Zupan , “4 Million Neonatal Deaths: When? Where? Why?,” Lancet 365, no. 9462 (2005): 891–900. 15752534 10.1016/S0140-6736(05)71048-5

[10] K. Robertsson Grossmann , M. Eriksson Westblad , M. Blennow , and K. Lindström , “Outcome at Early School Age and Adolescence After Hypothermia‐Treated Hypoxic–Ischaemic Encephalopathy: An Observational, Population‐Based Study,” Archives of Disease in Childhood. Fetal and Neonatal Edition 108, no. 3 (2023): 295–301. 36600485 10.1136/archdischild-2022-324418 PMC10176399

[11] B. L. Lee and H. C. Glass , “Cognitive Outcomes in Late Childhood and Adolescence of Neonatal Hypoxic‐Ischemic Encephalopathy,” Clinical and Experimental Pediatrics 64, no. 12 (2021): 608–618. 34044480 10.3345/cep.2021.00164 PMC8650814

[12] D. G. Calame and K. S. Fisher , “Improving Prognostication in Hypoxic‐Ischemic Encephalopathy,” JAMA Network Open 7, no. 12 (2024): e2449197. 39636643 10.1001/jamanetworkopen.2024.49197

[13] H. C. Glass , T. R. Wood , B. A. Comstock , et al., “Predictors of Death or Severe Impairment in Neonates With Hypoxic‐Ischemic Encephalopathy,” JAMA Network Open 7, no. 12 (2024): e2449188. 39636636 10.1001/jamanetworkopen.2024.49188 PMC11621987

[14] H. Pekeles , F. Al Amrani , M. Perez‐Morgui , P. Wintermark , and M. Shevell , “Characteristics of Children With Cerebral Palsy in the Post–Therapeutic Hypothermia Era,” Journal of Child Neurology 38, no. 3–4 (2023): 130–136. 36872628 10.1177/08830738231159162 PMC10226002

[15] R. Lee‐Kelland , S. Jary , J. Tonks , F. M. Cowan , M. Thoresen , and E. Chakkarapani , “School‐Age Outcomes of Children Without Cerebral Palsy Cooled for Neonatal Hypoxic–Ischaemic Encephalopathy in 2008–2010,” Archives of Disease in Childhood. Fetal and Neonatal Edition 105, no. 1 (2020): 8–13. 31036702 10.1136/archdischild-2018-316509

[16] G. Erdi‐Krausz , R. Rocha , A. Brown , et al., “Neonatal Hypoxic‐Ischaemic Encephalopathy: Motor Impairment Beyond Cerebral Palsy,” European Journal of Paediatric Neurology 35 (2021): 74–81. 34666231 10.1016/j.ejpn.2021.10.005

[17] T. J. Robb , J. Tonks , A. P. C. Spencer , et al., “Communication Skills in Children Aged 6–8 Years, Without Cerebral Palsy Cooled for Neonatal Hypoxic‐Ischemic Encephalopathy,” Scientific Reports 12, no. 1 (2022): 17757. 36272982 10.1038/s41598-022-21723-1 PMC9588000

[18] C. J. Edmonds , R. Cianfaglione , C. Cornforth , and B. Vollmer , “Children With Neonatal Hypoxic Ischaemic Encephalopathy (HIE) Treated With Therapeutic Hypothermia Are Not as School Ready as Their Peers,” Acta Paediatrica 110, no. 10 (2021): 2756–2765. 34160861 10.1111/apa.16002

[19] M. Eriksson Westblad , K. Löwing , K. R. Grossmann , M. Blennow , and K. Lindström , “Long‐Term Motor Development After Hypothermia‐Treated Hypoxic‐Ischaemic Encephalopathy,” European Journal of Paediatric Neurology 47 (2023): 110–117. 37862884 10.1016/j.ejpn.2023.10.003

[20] T. Debillon , L. Sentilhes , G. Kayem , et al., “Risk Factors for Unfavorable Outcome at Discharge of Newborns With Hypoxic‐Ischemic Encephalopathy in the Era of Hypothermia,” Pediatric Research 93, no. 7 (2023): 1975–1982. 36272997 10.1038/s41390-022-02352-w

[21] P. Baxter , “Markers of Perinatal Hypoxia‐Ischaemia and Neurological Injury: Assessing the Impact of Insult Duration,” Developmental Medicine and Child Neurology 62, no. 5 (2020): 563–568. 31872436 10.1111/dmcn.14421

[22] A. Pappas , S. Shankaran , S. A. McDonald , et al., “Cognitive Outcomes After Neonatal Encephalopathy,” Pediatrics 135, no. 3 (2015): e624–e634. 25713280 10.1542/peds.2014-1566 PMC4338321

[23] J. L. Y. Cheong , L. Coleman , R. W. Hunt , et al., “Prognostic Utility of Magnetic Resonance Imaging in Neonatal Hypoxic‐Ischemic Encephalopathy: Substudy of a Randomized Trial,” Archives of Pediatrics & Adolescent Medicine 166 (2012): 634–640. 22751877 10.1001/archpediatrics.2012.284

[24] M. Rutherford , L. A. Ramenghi , A. D. Edwards , et al., “Assessment of Brain Tissue Injury After Moderate Hypothermia in Neonates With Hypoxic–Ischaemic Encephalopathy: A Nested Substudy of a Randomised Controlled Trial,” Lancet Neurology 9, no. 1 (2010): 39–45. 19896902 10.1016/S1474-4422(09)70295-9 PMC2795146

[25] S. B. Trivedi , Z. A. Vesoulis , R. Rao , et al., “A Validated Clinical MRI Injury Scoring System in Neonatal Hypoxic‐Ischemic Encephalopathy,” Pediatric Radiology 47, no. 11 (2017): 1491–1499. 28623417 10.1007/s00247-017-3893-y PMC6219383

[26] L. C. Weeke , F. Groenendaal , K. Mudigonda , et al., “A Novel Magnetic Resonance Imaging Score Predicts Neurodevelopmental Outcome After Perinatal Asphyxia and Therapeutic Hypothermia,” Journal of Pediatrics 192 (2018a): 33–40.e2. 29246356 10.1016/j.jpeds.2017.09.043 PMC5743051

[27] S. Shankaran , S. A. McDonald , A. R. Laptook , et al., “Neonatal Magnetic Resonance Imaging Pattern of Brain Injury as a Biomarker of Childhood Outcomes Following a Trial of Hypothermia for Neonatal Hypoxic‐Ischemic Encephalopathy,” Journal of Pediatrics 167, no. 5 (2015): 987–993.e3. 26387012 10.1016/j.jpeds.2015.08.013 PMC4700815

[28] S. Shankaran , P. D. Barnes , S. R. Hintz , et al., “Brain Injury Following Trial of Hypothermia for Neonatal Hypoxic‐Ischaemic Encephalopathy,” Archives of Disease in Childhood. Fetal and Neonatal Edition 97, no. 6 (2012c): F398–F404. 23080477 10.1136/archdischild-2011-301524 PMC3722585

[29] C. O. Lew , E. Calabrese , J. V. Chen , et al., “Artificial Intelligence Outcome Prediction in Neonates With Encephalopathy (AI‐OPiNE),” Radiology: Artifical Intelligence 6 (2024): e240076. 10.1148/ryai.240076 PMC11427921 38984984

[30] Y. W. Wu , J. L. Wisnowski , H. C. Glass , et al., “Advancing Brain MRI as a Prognostic Indicator in Hypoxic‐Ischemic Encephalopathy,” Pediatric Research 95, no. 3 (2024): 587–589. 37696979 10.1038/s41390-023-02786-w

[31] E. Calabrese , Y. Wu , A. W. Scheffler , et al., “Correlating Quantitative MRI‐Based Apparent Diffusion Coefficient Metrics With 24‐Month Neurodevelopmental Outcomes in Neonates From the HEAL Trial,” Radiology 308, no. 3 (2023): e223262. 37698478 10.1148/radiol.223262 PMC10546287

[32] M. Machie , L. Weeke , L. S. de Vries , N. Rollins , L. Brown , and L. Chalak , “MRI Score Ability to Detect Abnormalities in Mild Hypoxic‐Ischemic Encephalopathy,” Pediatric Neurology 116 (2021): 32–38. 33412459 10.1016/j.pediatrneurol.2020.11.015 PMC8087244

[33] R. Guillet , A. D. Edwards , M. Thoresen , et al., “Seven‐ to Eight‐Year Follow‐Up of the CoolCap Trial of Head Cooling for Neonatal Encephalopathy,” Pediatric Research 71, no. 2 (2012): 205–209. 22258133 10.1038/pr.2011.30

[34] K. J. Hassell , M. Ezzati , D. Alonso‐Alconada , D. J. Hausenloy , and N. J. Robertson , “New Horizons for Newborn Brain Protection: Enhancing Endogenous Neuroprotection,” Archives of Disease in Childhood. Fetal and Neonatal Edition 100, no. 6 (2015): F541–F552. 26063194 10.1136/archdischild-2014-306284 PMC4680177

[35] D. G. Kleuskens , F. Gonçalves Costa , K. V. Annink , et al., “Pathophysiology of Cerebral Hyperperfusion in Term Neonates With Hypoxic‐Ischemic Encephalopathy: A Systematic Review for Future Research,” Frontiers in Pediatrics 9 (2021): 631258. 33604320 10.3389/fped.2021.631258 PMC7884860

[36] K. O. Yeates , H. G. Taylor , N. C. Walz , T. Stancin , and S. L. Wade , “The Family Environment as a Moderator of Psychosocial Outcomes Following Traumatic Brain Injury in Young Children,” Neuropsychology 24, no. 3 (2010): 345–356. 20438212 10.1037/a0018387 PMC2976589

[37] V. A. Anderson , S. A. Morse , C. Catroppa , F. Haritou , and J. V. Rosenfeld , “Thirty Month Outcome From Early Childhood Head Injury: A Prospective Analysis of Neurobehavioural Recovery,” Brain: A Journal of Neurology 127, no. Pt 12 (2004): 2608–2620. 15537678 10.1093/brain/awh320

[38] L. Bartha‐Doering , A. Gleiss , S. Knaus , M. T. Schmook , and R. Seidl , “Influence of Socioeconomic Status on Cognitive Outcome After Childhood Arterial Ischemic Stroke,” Developmental Medicine and Child Neurology 63, no. 4 (2021): 465–471. 33336807 10.1111/dmcn.14779 PMC7986130

[39] N. Marlow , D. Wolke , M. A. Bracewell , and M. Samara , “Neurologic and Developmental Disability at Six Years of Age After Extremely Preterm Birth,” New England Journal of Medicine 352, no. 1 (2005): 9–19. 15635108 10.1056/NEJMoa041367

[40] M. Feldmann , V. Rousson , T. D. Nguyen , et al., “Cognitive Outcome of Early School‐Aged Children Born Very Preterm Is Not Predicted by Early Short‐Term Amplitude‐Integrated Electroencephalography,” Acta Paediatrica 109, no. 1 (2020): 78–84. 31254357 10.1111/apa.14919

[41] L. Lacalle , M. L. Martínez‐Shaw , Y. Marín , and Y. Sánchez‐Sandoval , “Intelligence Quotient (IQ) in School‐Aged Preterm Infants: A Systematic Review,” Frontiers in Psychology 14 (2023): 1216825. 37560105 10.3389/fpsyg.2023.1216825 PMC10409487

[42] J. W. Gaynor , R. F. Ittenbach , M. Gerdes , et al., “Neurodevelopmental Outcomes in Preschool Survivors of the Fontan Procedure,” Journal of Thoracic and Cardiovascular Surgery 147, no. 4 (2014): 1276–1283.e5. 24521968 10.1016/j.jtcvs.2013.12.019 PMC5662937

[43] D. Huisenga , G. S. La Bastide‐Van , A. Van Bergen , J. Sweeney , and M. Hadders‐Algra , “Developmental Outcomes After Early Surgery for Complex Congenital Heart Disease: A Systematic Review and Meta‐Analysis,” Developmental Medicine and Child Neurology 63, no. 1 (2021): 29–46. 32149404 10.1111/dmcn.14512 PMC7754445

[44] J. R. Flynn , What Is Intelligence?: Beyond the Flynn Effect, 1st ed. (Cambridge, United Kingdom: Cambridge University Press, 2007), https://www.cambridge.org/core/product/identifier/9780511605253/type/book.

[45] K. B. Billeiter , J. M. Froiland , J. P. Allen , and D. B. Hajovsky , “Neurodiversity and Intelligence: Evaluating the Flynn Effect in Children With Autism Spectrum Disorder,” Child Psychiatry and Human Development 53, no. 5 (2022): 919–927. 33939111 10.1007/s10578-021-01175-w

