# Supplementary figures and images for: Predictors for Development of Asphyxiated Neonates Treated With Therapeutic Hypothermia
Source: Acta Paediatr. 2025 Jan 29;114(7):1553–61. doi: 10.1111/apa.17598 (PMC12147419; doi:10.1111/apa.17598)

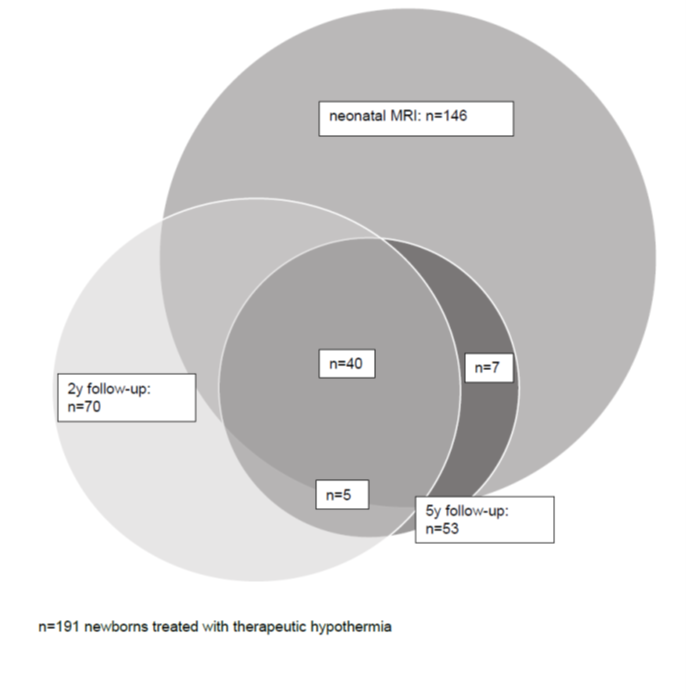

Supplement: Supplementary file 1 — Figure S1. [file APA-114-1553-s002.png]
