# Supplementary material for: Predictors for Development of Asphyxiated Neonates Treated With Therapeutic Hypothermia
Source: Acta Paediatr. 2025 Jan 29;114(7):1553–61. doi: 10.1111/apa.17598 (PMC12147419; doi:10.1111/apa.17598)
Supplement: Supplementary file 2 — Table S1. Table S2. [file APA-114-1553-s001.docx]

**Supplementary Table S1. Neonatal characteristics of cohort**

| **Parameter** | **Study cohort** | **n** | **Lost to FU** | **n** | **p** |
| --- | --- | --- | --- | --- | --- |
| Birthweight (g) | 3360 (2120 - 5175) | 51 | 3280 (1740 - 5620) | 111 | ns |
| Female (n) | 21 (40.38%) | 52 | 51 (45.54%) | 112 | ns |
| Gestational age (weeks) | 40.07 (35.14 - 41.86) | 52 | 39.22 (35.14 – 42.71) | 112 | ns |
| Min pH in first 60 min | 6.87 (6.53 - 7.21) | 49 | 6.9 (6.48 – 7.22) | 106 | ns |
| Min BE in first 60 min (mmol/l) | -21.9 ( -45.0 – 11.0) | 47 | -20.35 (-41.2 - -8.2) | 104 | ns |
| Apgar 5 min | 4 (0 - 9) | 50 | 4 (0 - 10) | 111 | ns |
| Time to hypothermia (min) | 182 (58 - 761) | 49 | 173 (15 - 648) | 108 | ns |
| Duration of hypothermia (h) | 73.33 (64.17-98.75) | 49 | 72.5 (3.5-77.50) | 109 | ns |

Data represent numbers (percentage) or median (range)

**Supplementary Table S2. Testing characteristics at 5-year follow-up**

|  |  |
| --- | --- |
| Age at follow-up (months), median (min - max) | 65 (58-72) |
| IQ score, median (min - max) | 103 (66-131) |
| IQ ≥ 115, n (%) | 9 (16.9) |
| IQ 85 - 114, n (%) | 24 (45.3) |
| IQ 60 - 84, n (%) | 10 (18.86) |
| Severely impaired (not testable), n (%) | 10 (18.86) |
